# Nephrolithiasis and Polycystic Ovary Syndrome: A Case-Control Study Evaluating Testosterone and Urinary Stone Metabolic Panels

**DOI:** 10.1155/2019/3679493

**Published:** 2019-10-17

**Authors:** Donald Fedrigon, Kareem Alazem, Sri Sivalingam, Manoj Monga, Juan Calle

**Affiliations:** Cleveland Clinic, Glickman Urological & Kidney Institute, 9500 Euclid Avenue, Cleveland, OH 44195, USA

## Abstract

**Introduction:**

Both elevated testosterone and polycystic ovary syndrome (PCOS) have been speculated as possible risk factors for kidney stone formation; however, the details of this potential relationship with regards to 24-hour urine metabolic panels and stone composition have not previously been characterized.

**Methods:**

A total of 74 PCOS patients were retrospectively identified and matched with a cohort of female stone formers at a 3 : 1 ratio (by age and BMI). All patients had 24-hour urinary metabolic panels and stone compositions. These groups were compared using Pearson chi-square and Student *t*-tests. Additionally, the PCOS group was differentiated based on free testosterone using multivariate analysis.

**Results:**

The case-control cohort showed that PCOS patients had significantly lower sodium excretion (*p*=0.015) and hypernatriuria rates (28.9% vs 50.9%, *p*=0.009). The PCOS-testosterone cohort demonstrated that high testosterone patients had significantly higher citrate values (*p*=0.041) and significantly lower odds of hypocitraturia (36.7% vs 54.2%, OR = 0.2, *p*=0.042). The high testosterone group also had higher sodium excretion (*p*=0.058) with significantly higher odds of having hypernatriuria (40.0% vs 13.6%, OR = 13.3, *p*=0.021). No significant patterns were revealed based on stone composition analysis.

**Conclusions:**

Compared to healthy stone formers, PCOS patients did not demonstrate significant differences in 24-hour urine and stone composition values. Elevated free testosterone in PCOS patients has a significant association with higher urinary citrate and sodium values: findings that in and of themselves do not confirm the hypothesized increased risk of stone formation. This patient cohort may provide deeper insight into the interplay between androgens and stone formation; however, further study is needed to fully characterize the possible relationship between PCOS and stone formation.

## 1. Introduction and Objective

Polycystic ovary syndrome (PCOS) is a common endocrine disorder characterized by oligomenorrhea and androgen excess, which affects 5–10% of women at reproductive-age and has been suggested to be a potential risk factor for stone formation [1, 2]. Elevated testosterone has been implicated as a causal factor in kidney stone formation based on a variety of inferential evidence: the incidence of urolithiasis is 2-3 times higher in men than in women, the risk of stone formation is reduced in castrated rats, as well as male stone formers having higher serum total testosterone levels than stone-free controls [3–8]. Our goal was to evaluate the relationship between PCOS, elevated testosterone, and stone formation from the perspective of stone composition and 24-hour urine metabolic stone panels.

## 2. Methods

### 2.1. Study Design

We retrospectively identified 74 patients from the Cleveland Clinic stone registry (ICD-9 592.xx and 594.xx) with diagnosis of PCOS (ICD-9 256.4) who had a recorded free testosterone level and 24-hour urinary collection. A matched cohort of female stone formers, matched by age and BMI on the date of 24-hour urine collection, was generated from our registry at a 3 : 1 ratio, for a total of 222 control patients. Patients were selected from our database in the timeframe from Oct. 2002 to Jul. 2014. If multiple testosterone values were available, the result closest in time to the date of the 24-urine collection was used. The PCOS patients were divided into normal and high (>6.4 pg/mL) testosterone groups based on previously published reference values [9].

### 2.2. Metabolic and Laboratory Evaluation

Stone composition was analyzed by Fourier transform infrared spectrometry and expressed as the amount of each component in the stone. Studied components were calcium oxalate monohydrate (CaOMH), calcium oxalate dihydrate (CaODH), calcium phosphate (CaPh), calcium hydrogen phosphate (CaHPh), magnesium ammonium phosphate (MAP), and uric acid (UA). A stone was considered pure if 70% or greater of the stone was composed of a single component. Patient urinary risk factors for stone disease were evaluated by 24-hour urine collection with the patient on an unrestricted diet. Urine samples were examined for calcium, sodium, oxalate, citrate, UA, volume, and creatinine. Urinary normal values were set using specific methodologies, including calcium less than 200 mg per day for women by the o-cresolphthalein complexone method, sodium less than 150 mmol per day by ion-selective electrode, oxalate less than 40 mg per day by enzyme immunoassay, citrate greater than 550 mg per day for women by enzyme immunoassay, UA less than 750 mg per day for women by the uricase method, and volume 2,000 ml or greater per day. Urinary creatinine was measured by the enzymatic method. Serum-free testosterone was evaluated by liquid chromatography tandem mass spectrometry (LC/MS/MS).

### 2.3. Study Endpoints

Primary endpoints for both sets of analysis were 24-hour urinary metabolic panels and stone composition. We compared baseline 24-hour urinary metabolic panels within both the case-control and PCOS-testosterone cohorts by absolute levels of all parameters and the proportion of patients with specific abnormalities. We compared stone composition by the relative amount of each component in the stone, mixed/pure status, and proportion of patients with each pure stone type. When multiple stones or stone fragments were passed or retrieved surgically for a single episode of nephrolithiasis, they were sent for analysis as a single sample.

### 2.4. Medications and Comorbidities

Registry patients were not included in either the PCOS or control group if their 24-hour urinary metabolic panel overlapped with medications such as thiazides, citrates, or allopurinol that could impact stone formation or urine metabolic panels. Medications commonly used in the treatment of PCOS (i.e., oral contraceptives, spironolactone, and metformin) and common comorbidities (i.e., diabetes mellitus and hypertension) were evaluated for potential confounding effects on free testosterone levels. Only metformin was found to have a potential confounding effect and was therefore included, along with age and BMI, as an adjusted variable in multivariable analysis.

### 2.5. Statistical Analysis

Statistical analysis was performed using R Statistical Software (R Foundation for Statistical Computing, Vienna, Austria). Case-control and PCOS testosterone group means were compared using Student *t*-tests and group percentages were compared using Pearson chi-square. Within the PCOS cohort, endpoints were also compared using multivariable linear and logistic regression, adjusted for age, BMI, and metformin status. For all tests, *p* < 0.05 was considered statistically significant.

## 3. Results

### 3.1. Urinary Stone Panel: Case-Control Cohort

We identified 88 PCOS patients in our stone registry, of which 14 were excluded based on medications that could impact 24-hour urine results. Demographic characteristics as well as the urinary calcium, citrate, creatinine, oxalate, uric acid, and volume were similar between the PCOS and control groups (Table 1). The 24-hour urine metabolic panels for the case-control cohort revealed that PCOS patients had both significantly lower urinary sodium excretion (*p*=0.015) and lower frequency of hypernatriuria (28.9% vs 50.9%, *p*=0.009). Urinary citrate was higher in the PCOS group (624.1 ± 361.9) than that in the control group (527.5 ± 317.8) but this did not reach statistical significance (*p*=0.08).

### 3.2. Urinary Stone Panel: PCOS Testosterone Cohort

Within the 74 eligible PCOS patients, 38 (51.3%) were classified as having high free testosterone (>6.4 pg/mL). Diabetes (*n* = 11), hypertension (*n* = 25), spironolactone (*n* = 8), and oral contraceptive (*n* = 16) status were all evaluated as potential confounding variables for both nominal and continuous testosterone values but not found to be statistically significant. High testosterone classification was significantly associated with younger age (*p*=0.008), higher BMI (*p* < 0.001), and active metformin therapy (*p*=0.05). PCOS patients on metformin also had significantly higher free testosterone values (*p*=0.026). Therefore, BMI, age, and metformin status were included as confounding variables in the multivariate analysis.  Table 2 shows the original data analysis and significance values for the PCOS testosterone cohort.

Multivariate analysis revealed that high testosterone patients had significantly higher urinary citrate values (*p*=0.041, Table 3) with significantly lower odds of having hypocitraturia (36.7% vs 54.2%, OR = 0.2, *p*=0.042). High testosterone PCOS patients also had higher urinary sodium that approached statistical significance (*p*=0.058) with significantly higher odds of having hypernatriuria (40.0% vs 13.6%, OR = 13.3, *p*=0.021). Additionally, high testosterone PCOS patients had a higher average urinary volume output (*p*=0.066) and a decreased frequency of low urine volume (63.0% vs 69.2%, *p*=0.079) but these did not reach statistical significance.

Multivariate analysis of the PCOS patients using continuous free testosterone values did not reveal any statistically significant associations between 24-hour urine and stone composition parameters and free testosterone levels.

### 3.3. Stone Composition

Stone composition was recorded when present (24/74 PCOS patients, 215/222 controls). Stone composition assessment for the case-control cohort showed calcium oxalate monohydrate as the predominant component in both the PCOS patients and the controls (45.4% and 38.1%, respectively, Table 1). Mixed stone frequency was similar between the groups being present in 45.8% of the PCOS patients and 43.7% of the controls. Calcium oxalate monohydrate was the most frequent pure stone type in the PCOS group, while calcium phosphate was the most frequent amongst controls. Overall, stone composition assessment for both the testosterone and case-control cohorts did not reveal any statistically significant patterns in stone composition for either stone content quantitative or stone type qualitative parameters.

## 4. Discussion

### 4.1. PCOS-Nephrolithiasis Relationship

Even though the etiology of PCOS is still unclear, its clinical associations with hypertension, insulin resistance, and abdominal obesity due to androgen excess have been successfully documented [10–13]. However, the potential clinical association with kidney stones is still unexplored. We attempted to characterize the 24-hour urine metabolic and stone composition parameters of PCOS via our case-control cohort. Only sodium showed significant variations between PCOS patients with stones and our control group. Of relevance to this observation, there is evidence that sex differences can impact osmotic regulation of vasopressin and renal sodium handling, most likely due to alterations in testosterone and estrogen levels [14]. While we cannot make any definitive conclusions based on these findings, the broad hormonal changes in PCOS may be driving these differences in sodium excretion; however, menstrual cycle phase can also impact these outcomes that were not able to account for in our sample.

Polycystic ovary syndrome has also been linked to increased rates of the metabolic syndrome with an estimated prevalence of 33–43% in PCOS patients and a statistically significantly positive trend with free testosterone levels but only before adjusting for BMI [15, 16]. The metabolic syndrome in turn has been linked to several urological disease processes including nephrolithiasis [17]. Additionally, abdominal adiposity is commonly associated with PCOS and obesity is a well-documented risk factor for kidney stone formation [12, 18, 19].

Both our data and these observations indicate that a potential relationship between PCOS and kidney stones may not be directly related to hyperandrogenism but rather the obesity, metabolic syndrome, and/or insulin resistance that are frequently associated with this syndrome. It seems that urinary metabolic and stone composition data on PCOS patients compared with stone forming controls for age and BMI are not sufficient to determine whether PCOS is an independent risk factor for stone formation. Further prospective data regarding the prevalence of kidney stones in PCOS patients and potential associations within that group for obesity and the metabolic syndrome may shed more light on the issue.

### 4.2. Testosterone-Nephrolithiasis Relationship

The association between PCOS and hyperandrogenism offers an opportunity to explore the relationship between testosterone and urolithiasis [2]. However, PCOS is also a complex endocrine disorder with a variety of clinical signs, including variations in hyperandrogenism, which makes it heterogenous and difficult to diagnose [10]. Therefore, the lack of significant differences in our case-control cohort may have been due to our sample including a mixture of elevated and normal testosterone PCOS patients. Future studies may choose to look at only a subset of actively hyperandrogenic or newly diagnosed PCOS patients to account for this variation. This clinical variability in androgen levels could be due to both variations in treatment or disease severity. In light of these variations, we also chose to examine the impact of testosterone values within our PCOS group while accounting for antiandrogenic and other medications used in the treatment of PCOS.

Previous studies on the associations between androgens and nephrolithiasis have reached divergent conclusions, possibly due to variations in use of total and/or free testosterone assays. Several investigators have reported that both free and total testosterone are elevated in stone forming patients [4, 7]. However, others reported that only total testosterone is elevated in stone-forming patients [5, 8]. Clouding the discussion further, one study reported an increased risk of stones with lower testosterone levels [20]. A single study found no association with testosterone [21]. Our endocrinologists only evaluate free testosterone due to previous evidence that it is more sensitive than total testosterone for the diagnosis of hyperandrogenic disorders such as PCOS [22]. Our cutoff for high free testosterone classification was set at >6.4 pg/mL due to previously published reference ranges [9].

### 4.3. Testosterone: Citrate, Natriuria, and Urinary Volume

High free testosterone classification was associated in our multivariable analysis with increased urinary citrate, sodium, and volume (Table 1). Regarding the increase in citrate excretion, previous literature has found that PCOS patients have increased rates of cortisol production as well as insulin-resistance-related increase in serum aldosterone [23, 24]. These two processes may be contributing to a metabolic alkalosis leading in turn to the increased citrate excretion we observed; however, we were not able to obtain a large enough sample of serum bicarbonate values to fully examine this possibility. Higher citrate and urinary volumes have been found to be protective against the formation of kidney stones [25]. This may support a prior study that reported increased urinary stone risk in those with lower testosterone levels [20].

Previous examinations into testosterone's role in hypertension have indicated that testosterone may be driving a rightward shift in the pressure-natriuresis curve as well as contributing to increased interglomerular pressure [26]. Based on these findings, the respective increases in urine volume and sodium excretion among high testosterone PCOS patients could be secondary to this androgen-induced increase in interglomerular pressure and rightward shift in the pressure-natriuresis relationship.

### 4.4. Testosterone: Oxalate

Population data have shown that men have higher urinary oxalate excretion, an important promoter of lithogenesis [27]. Lee and colleagues noted decreased calcium oxalate stone formation in castrated male rats [3]. More recently, Nath, et al. reported significantly higher 24-hour urinary oxalate excretion in male stone formers and a positive association between free testosterone levels and urinary oxalate [4]. Despite these findings, we did not observe any significant changes in oxalate excretion within 24-hour urine metabolic panels or an increased frequency of calcium oxalate stones based on either PCOS or testosterone status.

### 4.5. Limitations

One major limitation to our study is its retrospective nature; however, a case-control methodology increases the likelihood that a difference in outcomes would be the result of PCOS. We also were not able to adjust for dietary factors that could impact 24-hour urine results or menstrual cycle timing that could impact free testosterone levels. It is also possible that the impact of androgens on stone risk in women may differ from that in men. Additionally, very few of the PCOS patients selected from the registry had stone composition analysis performed, which limited our analysis. Also, as a registry-based study, there is the possibility of bias regarding the criteria applied by different providers in assigning the diagnosis of polycystic ovarian syndrome.

## 5. Conclusions

When compared to matched cohort of healthy stone formers, PCOS patients did not demonstrate significant differences in 24-hour urine and stone composition values. It is possible that the potential relationship between PCOS and kidney stones may not be directly related to hyperandrogenism but rather the obesity, metabolic syndrome, and/or insulin resistance that are frequently associated with this syndrome. Elevated free testosterone in PCOS patients demonstrated a significant association with higher sodium and urine volume, possibly secondary to an increased intraglomerular pressure and a rightward shift in the pressure-natriuresis relationship. This patient cohort may provide deeper insight into the interplay between androgens and stone formation; however, further study is needed to fully characterize the possible relationship between PCOS and stone formation.

## Figures and Tables

**Table 1:** Bivariate analysis using PCOS status within case-control cohort.

	PCOS (*n* = 74)		No PCOS (*n* = 222)		*p* value	*N*
Mean ± SD age (range)	35.9 ± 11.8	(13–73)	36.0 ± 11.7	(13–73)	0.989	296
Mean ± SD kg/m^2^ BMI (range)	31.2 ± 7.4	(17.9–54.5)	31.0 ± 7.3	(18.6–55.0)	0.865	296
Mean ± SD 24 hr urine panel (range):						
Calcium (mg/day)	211.2 ± 128.3	(35.5–913.6)	214.7 ± 119.7	(16.7–817.1)	0.848	251
Citrate (mg/day)	624.1 ± 361.9	(82–1903)	527.5 ± 317.8	(4–1546)	0.083	220
Creatinine (g/day)	1.3 ± 0.4	(0.6–3.2)	1.3 ± 0.41	(0.1–2.9)	0.316	230
Oxalate (mg/day)	38.4 ± 15.2	(17–113)	39.1 ± 20.8	(0–180)	0.777	224
Sodium (mmol/day)	135.7 ± 52.0	(49–283)	158.8 ± 76.3	(28–442)	0.015	211
Uric acid (mg/day)	520.2 ± 194.6	(179–1016)	550.1 ± 210.2	(30.3–1361.3)	0.319	231
Volume (ml/day)	1643 ± 793.6	(581–3440)	1690 ± 789.9	(310–4450)	0.712	188
No. abnormality (%):						
Hypercalciuria (>200 mg/day)	35	(53.9%)	87	(46.8%)	0.402	251
Hypocitraturia (<550 mg/day)	24	(44.4%)	95	(57.2%)	0.139	220
Hyperoxaluria (>40 mg/day)	20	(34.5%)	58	(34.9%)	1.000	224
Hypernatriuria (>150 mmol/day)	15	(28.9%)	81	(50.9%)	0.009	211
Hyperuricosuria (>750 mg/day)	8	(13.3%)	29	(17.0%)	0.650	231
Low volume (<2000 ml/day)	35	(66.0%)	93	(68.9%)	0.839	188
Mean ± SD stone composition % (range):						
CaOMH	45.4 ± 31.2	(0–90)	38.1 ± 31.4	(0–90)	0.286	239
CaODH	16.9 ± 19.0	(0–60)	17.0 ± 20.4	(0–90)	0.985	239
CaPh	25.2 ± 29.0	(0–90)	32.4 ± 34.6	(0–100)	0.270	239
CaHPh	4.6 ± 17.2	(0–80)	2.0 ± 11.7	(0–90)	0.479	239
MAP	2.5 ± 12.3	(0–60)	3.2 ± 12.9	(0–70)	0.791	239
UA	1.7 ± 4.8	(0–20)	3.4 ± 15.9	(0–100)	0.240	239
No. greater than 70% mixed (%)^*∗*^	11	(45.8%)	94	(43.7%)	1.000	239
No. 70% or greater pure (%)^*∗*^					0.261	239
CaOMH	9	(69.2%)	48	(39.7%)		
CaODH	0	(0%)	8	(6.6%)		
CaPh	3	(23.1%)	54	(44.6%)		
CaHPh	1	(7.7%)	3	(2.5%)		
MAP	0	(0%)	2	(1.7%)		
UA	0	(0%)	6	(5.0%)		

^*∗*^Stones qualified as pure if the dominant component made up ≥ 70% of the stone.

**Table 2:** Bivariate analysis using categorical testosterone levels within the PCOS cohort.

	High testosterone (*n* = 38)		Normal testosterone (*n* = 36)		*p* value	*N*
Mean ± SD age (range)	32.4 ± 8.2	(14.9–57.0)	39.7 ± 13.8	(13.63–73.69)	0.008	74
Mean ± SD kg/m^2^ BMI (range)	34.3 ± 7.0	(21.0–54.5)	27.9 ± 6.3	(17.93–48.79)	<0.001	74
Mean ± SD 24 hr urine panel (range)						
Calcium (mg/day)	223.7 ± 158.5	(35.5–913.6)	198.3 ± 87.7	(50.5–366.7)	0.426	65
Citrate (mg/day)	709.6 ± 411.7	(118–1903)	517.1 ± 257.9	(82–956	0.041	54
Creatinine (g/day)	1.3 ± 0.5	(0.6–3.2)	1.2 ± 0.3	(0.6–1.6)	0.224	56
Oxalate (mg/day)	37.6 ± 12.3	(17–66)	39.3 ± 18.3	(19–113)	0.681	58
Sodium (mmol/day)	144.4 ± 54.1	(49–282)	123.8 ± 47.8	(52–283)	0.154	52
Uric acid (mg/day)	565.3 ± 225.2	(179–1016)	461.4 ± 126.9	(208–739)	0.028	60
Volume (ml/day)	1721.8 ± 822.9	(581–3440)	1561.1 ± 769.6	(590–3259)	0.466	53
No. abnormality (%)						
Hypercalciuria (>200 mg/day)	17	(51.5%)	18	(56.3%)	0.893	65
Hypocitraturia (<550 mg/day)	11	(36.7%)	13	(54.2%)	0.312	54
Hyperoxaluria (>40 mg/day)	13	(41.9%)	7	(25.9%)	0.316	58
Hypernatriuria (>150 mmol/day)	12	(40.0%)	3	(13.6%)	0.078	52
Hyperuricosuria (>750 mg/day)	8	(23.5%)	0	(0%)	0.008	60
Low volume (<2000 ml/day)	17	(63.0%)	18	(69.2%)	0.848	53
Mean ± SD stone composition % (range)						
CaOMH	35.7 ± 31.8	(0–80)	59.0 ± 26.0	(0–90)	0.062	24
CaODH	16.4 ± 17.8	(0–60)	17.5 ± 21.5	(0–50)	0.899	24
CaPh	31.4 ± 31.8	(0–90)	16.5 ± 23.3	(0–80)	0.199	24
CaHPh	7.9 ± 22.3	(0–80)	—	—	0.209	24
MAP	4.3 ± 16.0	(0–60)	—	—	0.336	24
UA	0.7 ± 2.7	(0–10)	3.0 ± 6.8	(0–20)	0.332	24
No. greater than 70% mixed (%)^*∗*^	7	(50%)	4	(40%)	1.000	24

No adjustments made for confounding variables, original analysis only, and therefore, *p* values not referenced in discussion. ^*∗*^Stones qualified as pure if the dominant component made up ≥70% of the stone.

**Table 3:** Multivariate analysis using categorical testosterone levels within the PCOS cohort.

Linear regression	*β* coefficient	Standard error	*p* value	*N*

Mean ± SD 24 hr urine panel (range):				
Calcium (mg/day)	−32.4	38.8	0.406	65
Citrate (mg/day)	−246.3	117.4	0.041	54
Creatinine (g/day)	−0.1	0.1	0.691	56
Oxalate (mg/day)	1.8	4.8	0.705	58
Sodium (mmol/day)	−36.7	18.9	0.058	52
Uric acid (mg/day)	−28.4	57.2	0.621	60
Volume (ml/day)	−449.5	238.7	0.066	53
Mean ± SD stone composition % (range):				
CaOMH	11.9	13.6	0.392	24
CaODH	3.6	10.9	0.743	24
CaPh	2.5	12.9	0.851	24
CaHPh	−14.4	9.5	0.144	24
MAP	−9.6	6.3	0.145	24
UA	1.8	2.4	0.481	24

Logistic regression	Odds ratio	95% CI	*p* value	*N*

No. abnormality (%):				
Hypercalciuria (>200 mg/day)	1.0	0.32–3.33	0.955	65
Hypocitraturia (<550 mg/day)	0.2	0.04–0.94	0.042	54
Hyperoxaluria (>40 mg/day)	1.9	0.50–7.14	0.340	58
Hypernatriuria (>150 mmol/day)	13.3	1.49–100	0.021	52
Hyperuricosuria (>750 mg/day)	6250	4 × 10^22^–9 × 10^−16^	0.693	60
Low volume (<2000 ml/day)	0.2	0.04–1.19	0.079	53
No. greater than 70% mixed (%)^*∗*^	1.0	0.11–9.09	0.978	24

Note: confounding variables adjusted for age, BMI, and metformin status with high or normal testosterone classification as an independent variable. Odds ratio is reported as the likelihood of a high testosterone PCOS patient having a given urine or stone analysis finding. Negative *β* coefficient indicates an elevation of that parameter in the high testosterone cohort. CI = confidence interval.

## Data Availability

Due to the policies of our institutional review board, we are unable to share the data obtained via our kidney stone registry with researchers outside of our institution at this time.

## Abbreviations

CaOMH: Calcium oxalate monohydrate
CaODH: Calcium oxalate dihydrate
CaPh: Calcium phosphate
CaHPh: Calcium hydrogen phosphate
CI: Confidence interval
LC/MS/MS: Liquid chromatography tandem mass spectrometry
MAP: Magnesium ammonium phosphate
PCOS: Polycystic ovarian syndrome
UA: Uric acid.
